# The Zen Garden Virtual Reality App for eating disorders: description and preliminary results

**DOI:** 10.1192/j.eurpsy.2024.260

**Published:** 2024-08-27

**Authors:** D. Gravina, S. Kirupakaran, M. Badr, A. Meaburn, M. Kresojevic, L. Dell’Osso, J. Treasure, H. Himmerich

**Affiliations:** ^1^Institute of Psychiatry, Psychology and Neuroscience, King’s College London, Centre for Research in Eating and Weight Disorders, London, United Kingdom; ^2^University of Pisa, Department of Clinical and Experimental Medicine, Pisa, Italy; ^3^Digital Innovation and Technology Services, South London and Maudsley NHS Foundation Trust; ^4^Maudsley Digital Lab; ^5^Bethlem Royal Hospital, South London and Maudsley NHS Foundation Trust, London, United Kingdom

## Abstract

**Introduction:**

Virtual Reality (VR) represents an emerging and promising tool to enhance standard care for patients with eating disorders (EDs). Indeed, VR provides an immersive and interactive experience in a safe and controlled environment that can simulate real-life situations, showing encouraging findings on various components of psychological treatments such as exposure therapy, psychoeducation, and emotional regulation.

**Objectives:**

This study aims to evaluate the Zen Garden VR App in patients with anorexia nervosa (AN) in order to obtain pilot data regarding changes in mood, relaxation, anger, anxiety, and weight and shape concerns. A secondary aim was to receive feedback from participants about the VR experience, its components, and its possible application for people with AN.

**Methods:**

Self-reported baseline and post-intervention data were collected from a sample of six female inpatients with AN recruited at the Eating Disorders Service at the Bethlem Royal Hospital of the South London and Maudsley NHS Foundation trust (SLaM). The technology used during the VR session consisted of an Oculus head-mounted display headset and two controllers which provided continuous rotational and positional tracking (Figures 1, 2 and 3).

**Results:**

Findings showed a global improvement after the VR Zen Garden App session, mainly in reducing levels of anxiety (Cohen’s d= 1.07) and promoting relaxation (Cohen’s d= 0.95), with possible applications especially before and after meals when food fears are at their highest. In addition, the music that was played during the intervention had a particularly positive effect.

**Image:**

**Figure:**
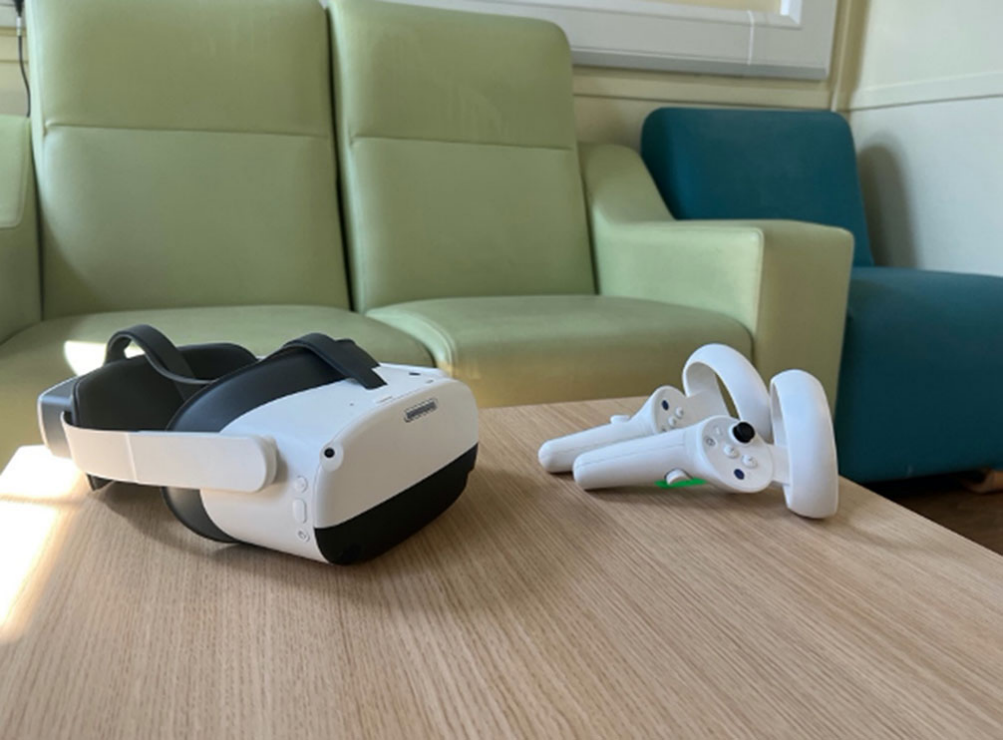

**Image 2:**

**Figure:**
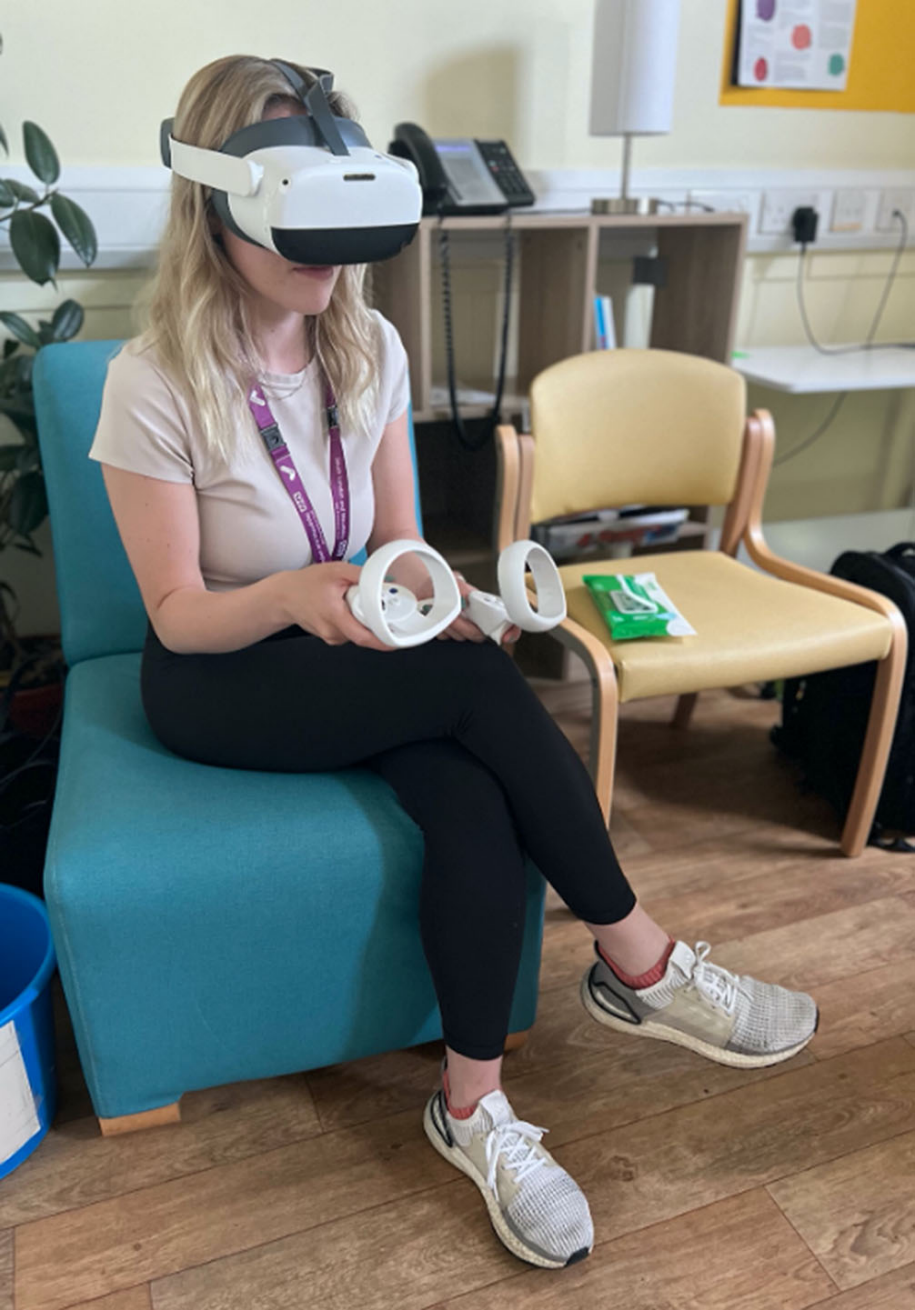

**Image 3:**

**Figure:**
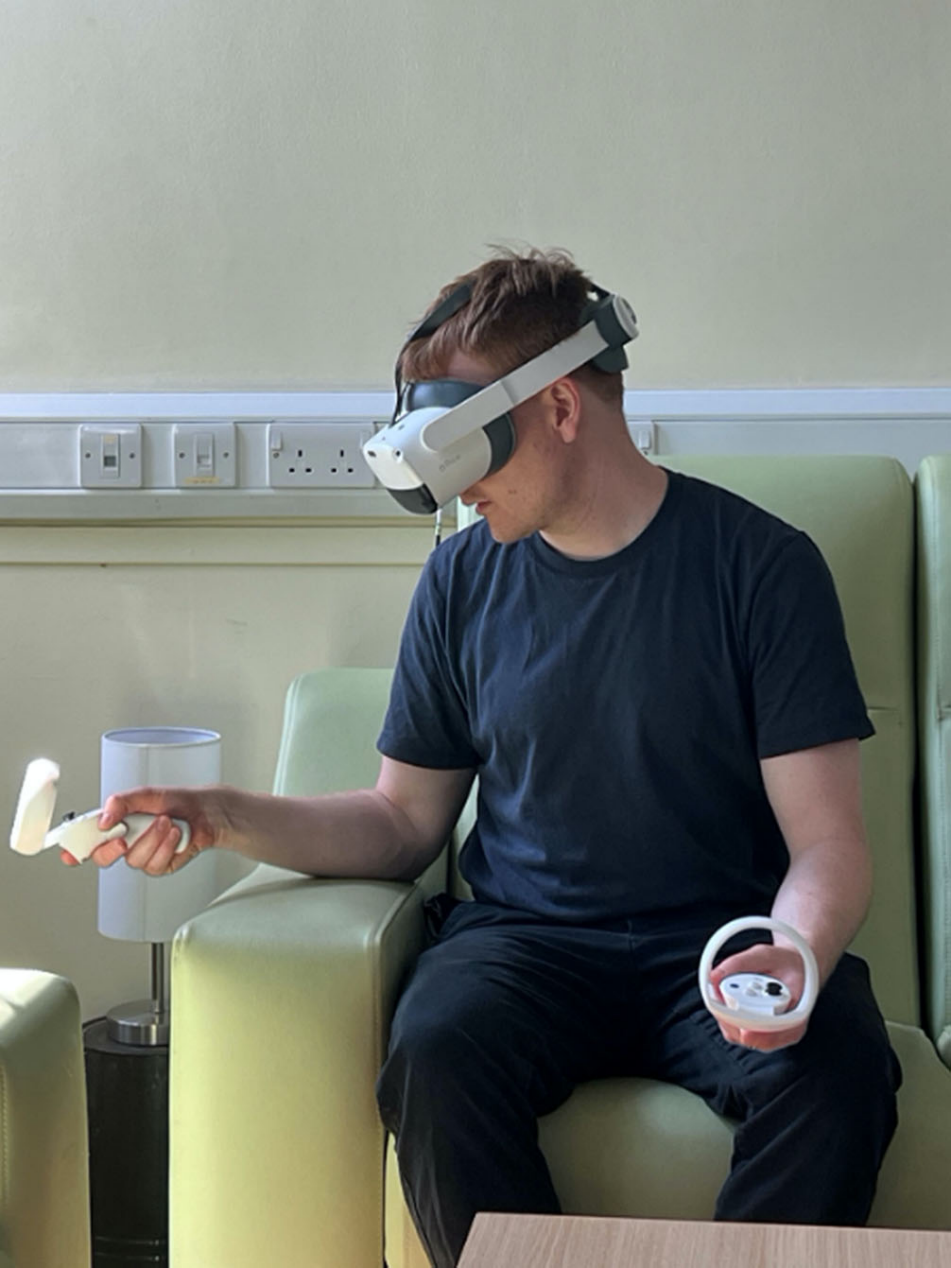

**Conclusions:**

Despite limitations, such us the small sample size and the one time point measurement, positive clinical implications have been highlighted for the Zen Garden VR App in patients with AN, although further studies are needed to confirm these preliminary findings. It is possible that VR could usefully augment and personalize care for people with an EDs. A range of interventions might be used to target the most compromised symptoms such as designing interventions that can help with triggers to eating disorder psychopathology.

**Disclosure of Interest:**

None Declared

